# Supplementary material for: Transcranial ultrasound stimulation selectively enhances fronto-temporal context-guided memory
Source: bioRxiv. 2025 Dec 22:2025.12.19.695543. Preprint. [Version 1] doi: 10.64898/2025.12.19.695543 (PMC12776154; doi:10.64898/2025.12.19.695543)
Supplement: Supplement 1 [file NIHPP2025.12.19.695543v1-supplement-1.pdf]

bioRxiv preprint doi: https://doi.org/10.64898/2025.12.19.695543; this version posted December 22, 2025. The copyright holder for this preprint (which was not certified by peer review) is the author/funder, who has granted bioRxiv a license to display the preprint in perpetuity. It is made available under aCC-BY 4.0 International license.

SUPPLEMENTARY INFORMATION

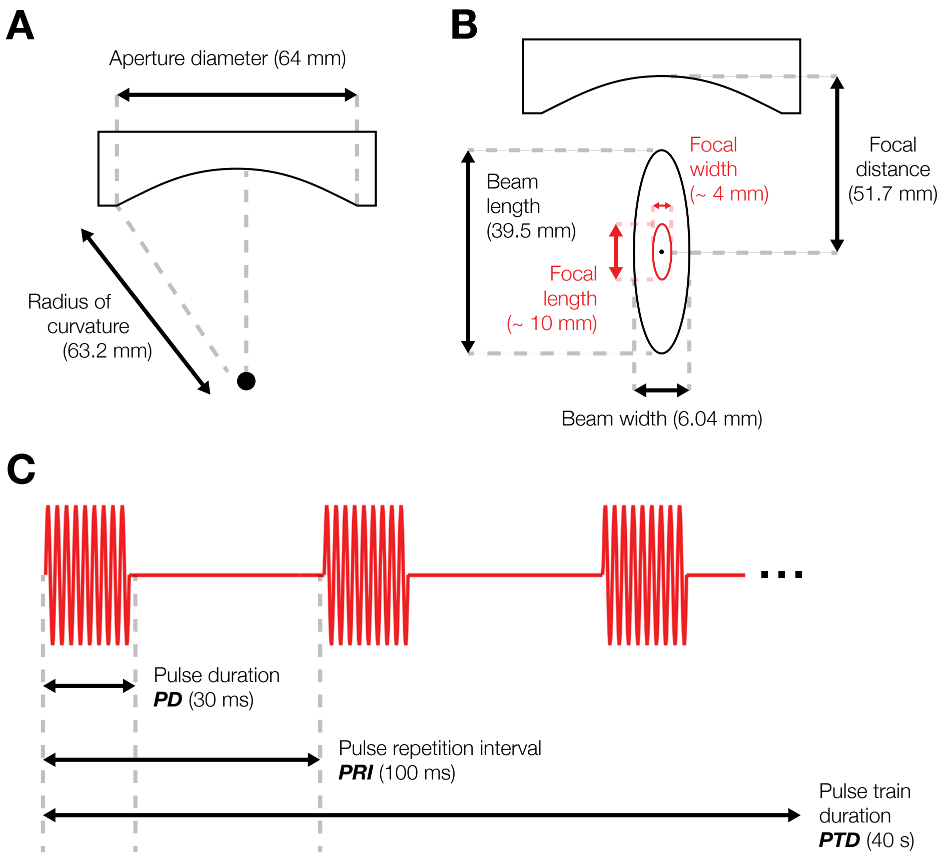

Supplementary Figure 1. Transducer dimensions and waveform parameters as outlined by ITRUSST.
A) Diameter of the transducer, and **B)** parameters of the focal distance, beam length, and beam width. **C)** Schematic of the ultrasound pressure waveform.

bioRxiv preprint doi: https://doi.org/10.64898/2025.12.19.695543; this version posted December 22, 2025. The copyright holder for this preprint (which was not certified by peer review) is the author/funder, who has granted bioRxiv a license to display the preprint in perpetuity. It is made available under aCC-BY 4.0 International license.

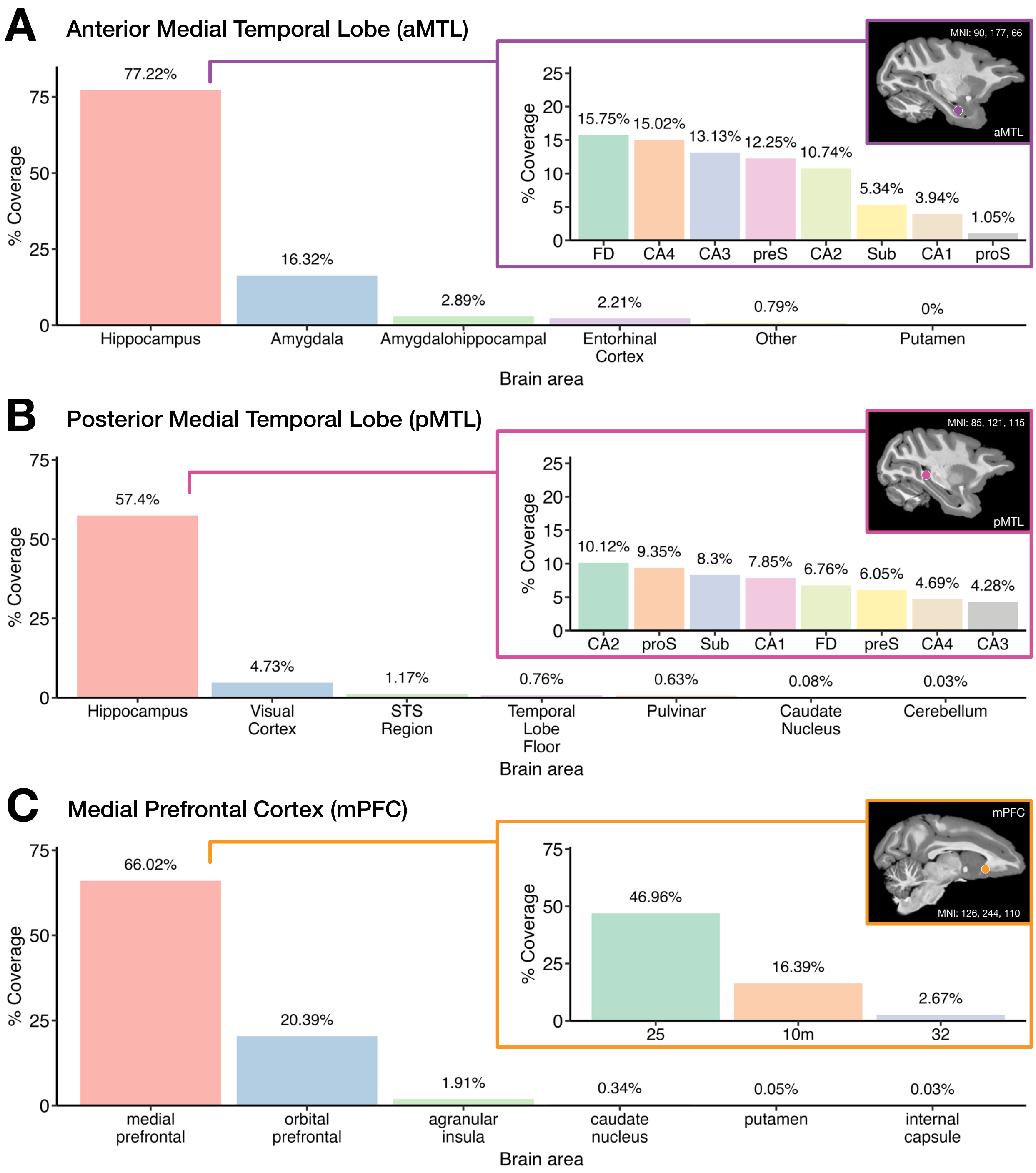

Supplementary Figure 2. Brain regions targeted by ultrasound stimulation and their corresponding coverage in the focal ellipsoid beam modelled using the D99 atlas brain.
The panels illustrate the percentage coverage of the ultrasound focal ellipsoid beam (L:10, W: 4, H: 4 mm) when targeting the three brain areas: **A)** Anterior hippocampus (aHPC), **B)** Posterior hippocampus (pHPC), and **C)** Medial prefrontal cortex (mPFC). Subplots within each panel focus on the subregions of the largest area affected. For **A-B)** this was the hippocampus, and **C)**, the medial prefrontal cortex.
